# Supplementary material for: Whole genome bisulfite sequencing of cell-free DNA and its cellular contributors uncovers placenta hypomethylated domains
Source: Genome Biol. 2015 Apr 15;16(1):78. doi: 10.1186/s13059-015-0645-x (PMC4427941; doi:10.1186/s13059-015-0645-x)
Supplement: Additional file 1: — Additional Supplementary Information. This file contains all supplementary figures referred to within the text of the manuscript. [file 13059_2015_645_MOESM1_ESM.docx]

**Supplementary Information. Jensen TJ, et al. Whole Genome Bisulfite Sequencing of Cell Free DNA and its Cellular Contributors Uncovers Placenta Hypomethylated Domains**

**
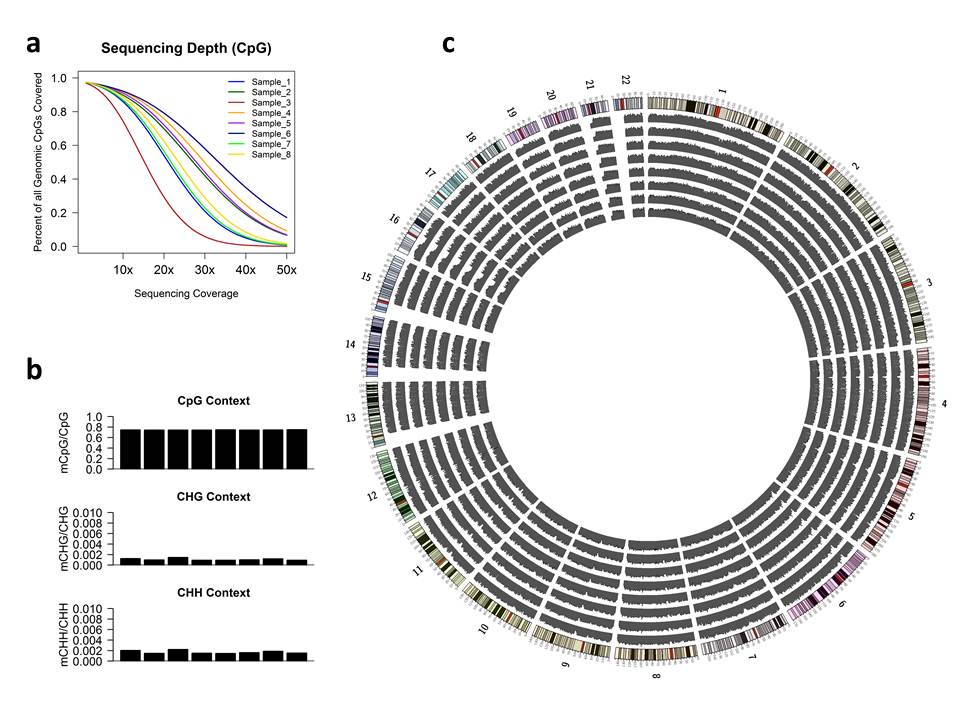
**

**Supplementary Figure 1. Characterization of the ccf DNA methylome. a)** Fraction of total genomic CpG sites covered at a particular depth. Each line denotes an individual sample. Y-axis represents the total portion of all genomic CpG sites which are covered at a corresponding depth (x-axis). **b)** Methylation level by cytosine context. Fraction of all cytosines which were methylated relative to the total number of measured cytosines are described for CpG, CHG, and CHH contexts. Each bar represents a single sample. **c)** Circos[^22^](#_ENREF_22) plot of all 8 non-pregnant ccf DNA samples. All autosomes are plotted and labeled along the perimeter with each concentric circle representing an individual sample. Height of each histogram represents the mean methylation level for CpG sites within the non-overlapping, 1MB bins.

**Note:** Supplementary Figure 2 is an independent file for easier viewing.

**Supplementary Figure 2. Circos plot of all sample types**[**^22^**](#_ENREF_22)**.** All autosomes are plotted and labeled along the perimeter with each concentric circle representing the mean methylation for each sample type. From the outside of the concentric circles working inward, methylation levels are shown for placenta (red), pregnant ccf DNA (blue), non-pregnant ccf DNA (green), and buffy coat (orange). The height of each histogram within each concentric circcle represents the mean methylation level for CpG sites within non-overlapping, 1MB genomic bins.


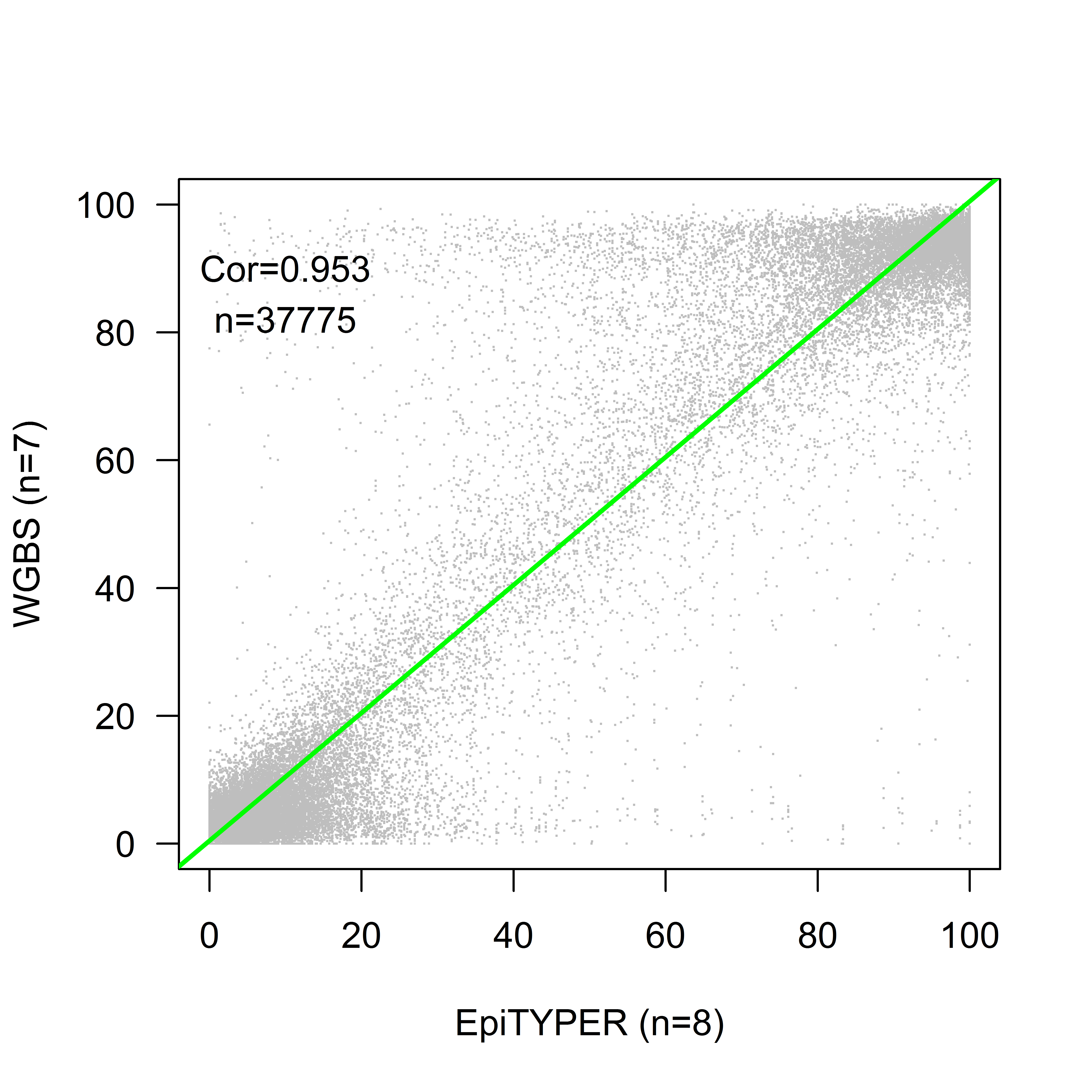


**Supplementary Figure 3.** Mean methylation for each CpG site (n=37775) as measured by EpiTYPER using MassARRAY (n=8; x-axis) and WGBS (n=7; y-axis) in two distinct sample cohorts of buffy coat. Each point represents a single CpG site; green line is a linear regression of the data; Cor=Pearson correlation coefficient.

**
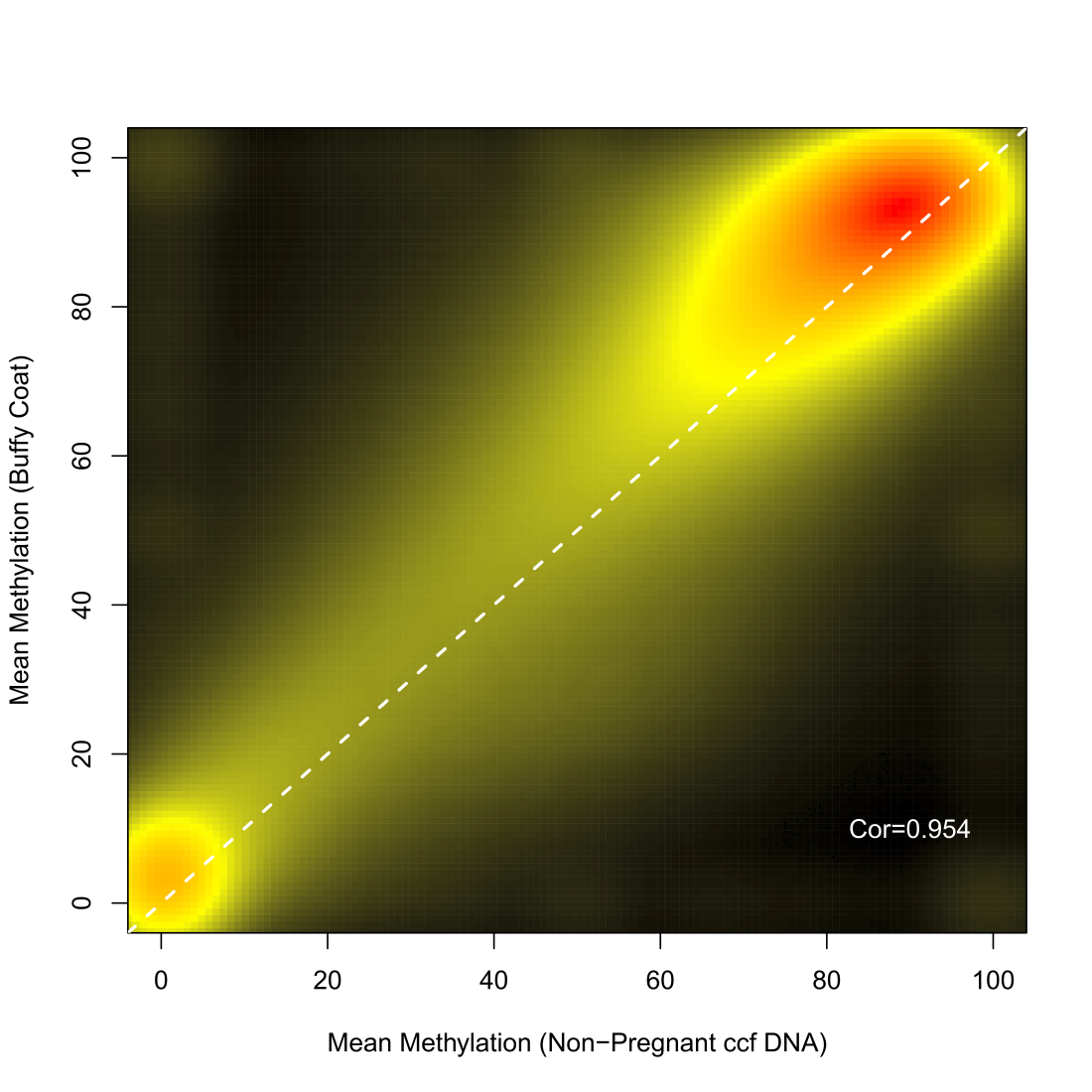
**

**Supplementary Figure 4. Correlation of CpG Methylation in Buffy Coat and non-pregnant ccf DNA.** Shown is a false color representation indicating the density of data points when comparing each paired CpG site in buffy coat (y-axis) and non-pregnant ccf DNA (x-axis). Black=low density, yellow=moderate density, red=high density. Cor=Pearson correlation coefficient.

**
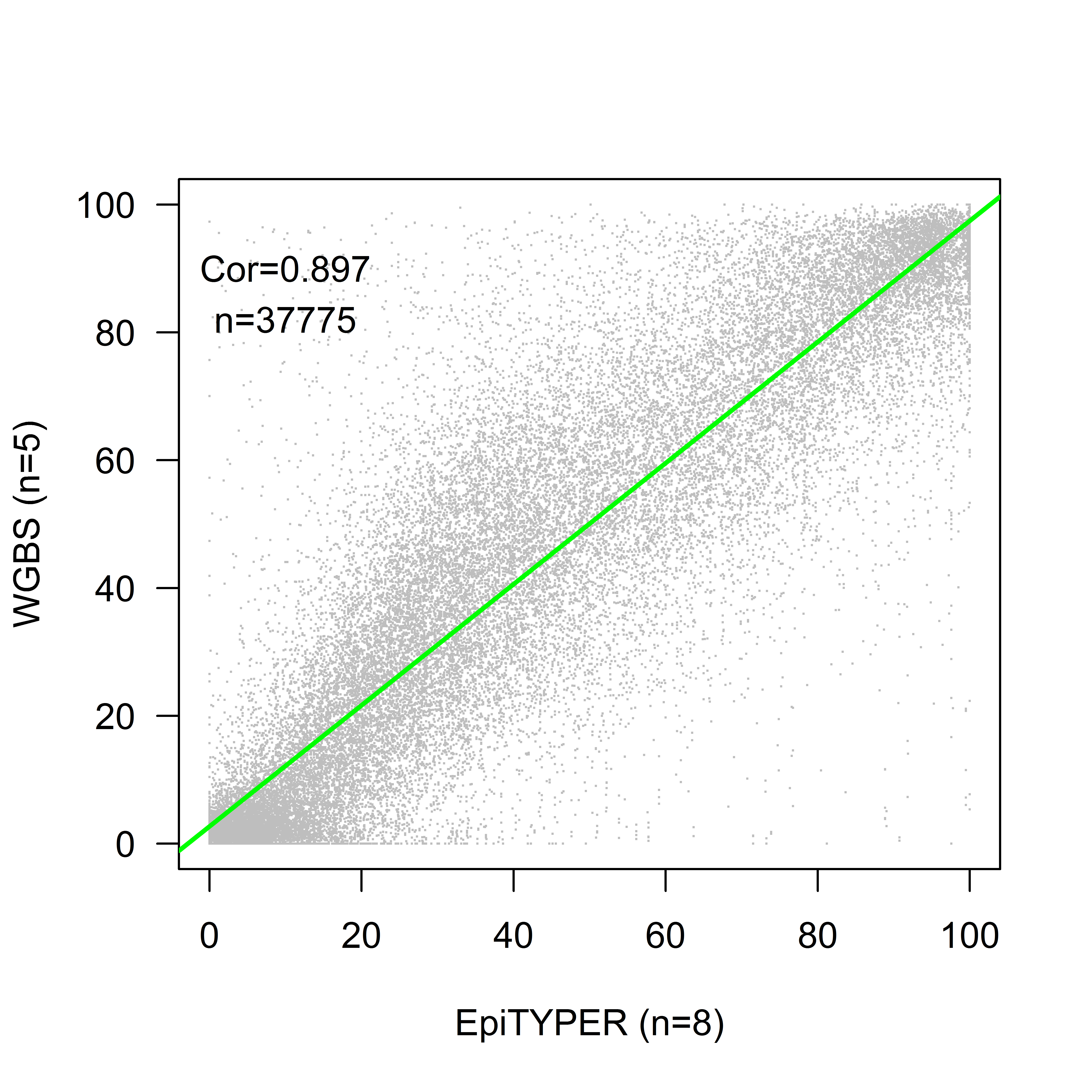
**

**Supplementary Figure 5.** Mean methylation for each CpG site (n=37775) as measured by EpiTYPER (n=8; x-axis) and WGBS (n=5; y-axis) in two distinct sample cohorts of placenta. Each point represents a single CpG site; green line is linear regression upon the data; Cor=Pearson correlation coefficient.

**
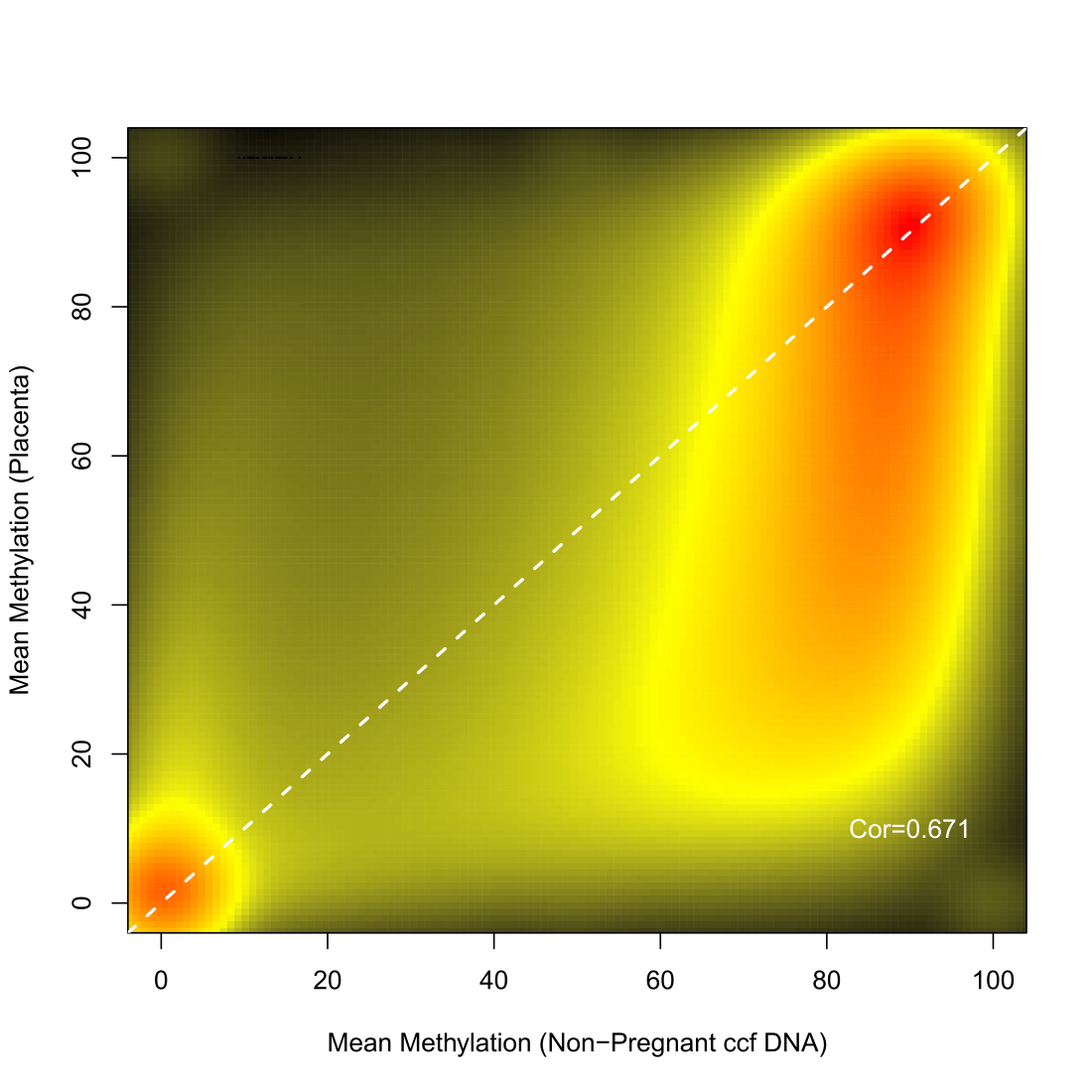
**

**Supplementary Figure 6. Correlation of CpG Methylation in Placenta and non-pregnant ccf DNA.** Shown is a false color representation indicating the density of data points when comparing each paired CpG site when in placenta (y-axis) and non-pregnant ccf DNA (x-axis). Black=low density, yellow=moderate density, red=high density. Cor=Pearson correlation coefficient.

**
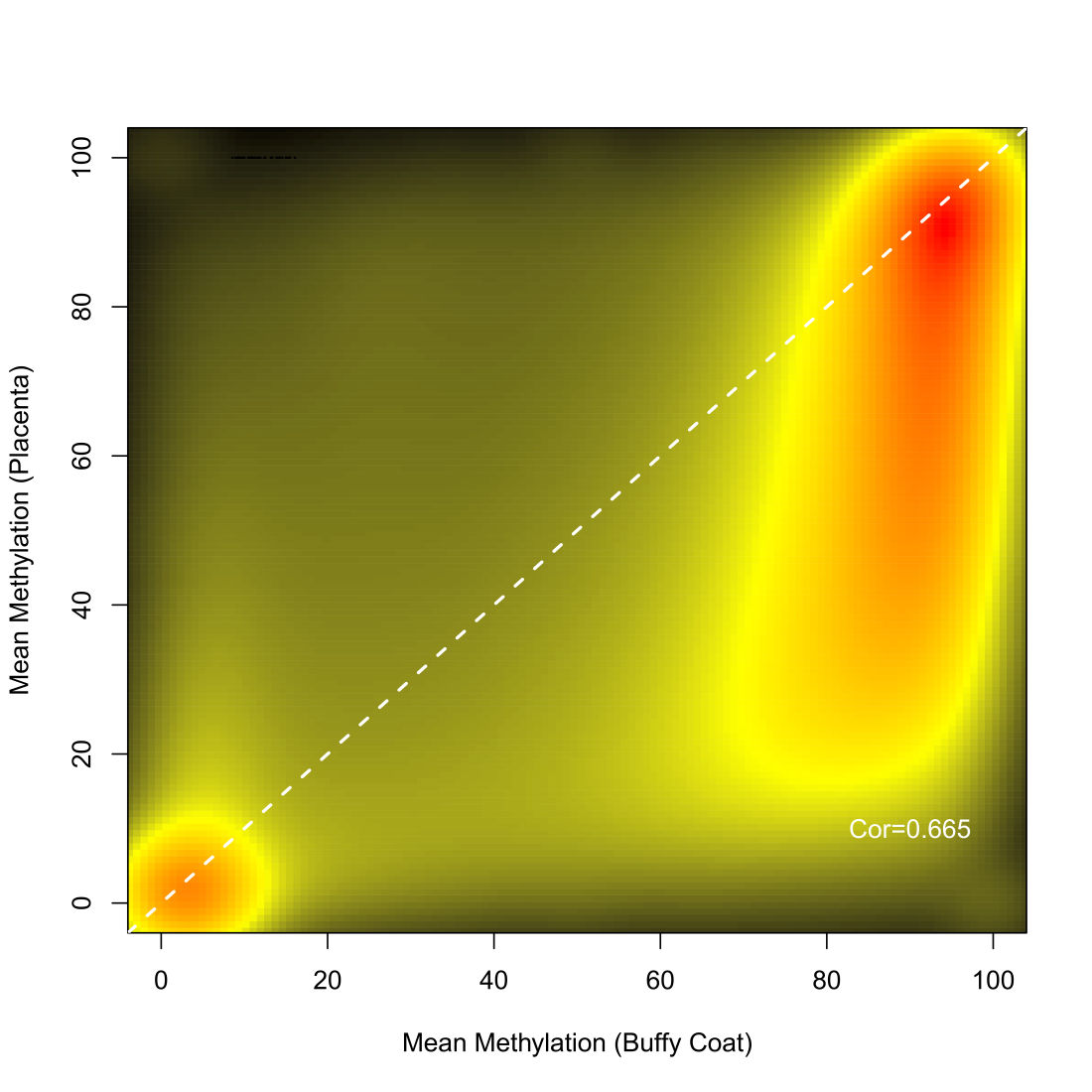
**

**Supplementary Figure 7. Correlation of CpG Methylation in Placenta and buffy coat DNA.** Shown is a false color representation indicating the density of data points when comparing each paired CpG site when in placenta (y-axis) and non-pregnant ccf DNA (x-axis). Black=low density, yellow=moderate density, red=high density. Cor=Pearson correlation coefficient.

**
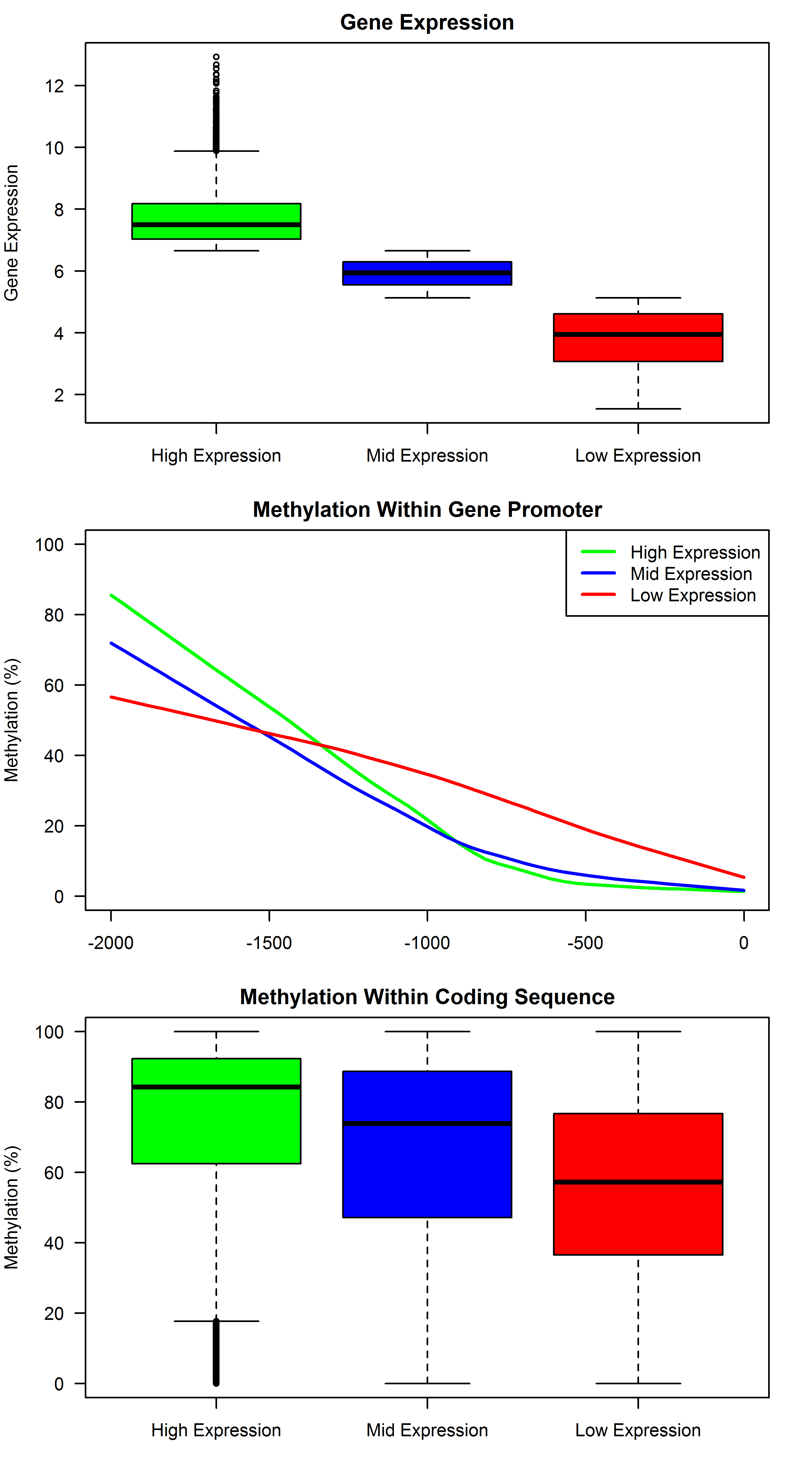
**

**Supplementary Figure 8. Placenta Gene Expression is linked to DNA methylation.** Gene expression analysis was performed on an independent cohort of 8 placenta samples using Affymetrix Human Exon 1.0 ST microarrays. Expression level was measured at the whole gene level for a total of 16231 genes. Genes were ranked and separated by expression levels with groups created from the top 33% (5410; green), middle 33% (5410 genes; blue), and bottom 33% (5411 genes; red) (top panel). Methylation levels were assessed within the promoters of each of the genes (middle panel). Methylation level was calculated within the coding sequence of all analyzed genes (bottom panel).





**Supplementary Figure 9. Description of genomic location of DMRs when comparing non-pregnant ccf DNA to placenta.** Each line represents the length of the corresponding chromosome. Regions more methylated in placenta (red) and buffy coat/PBMC (blue) shown. Distance from horizontal line for each chromosome is indicative of magnitude of differential methylation.

**
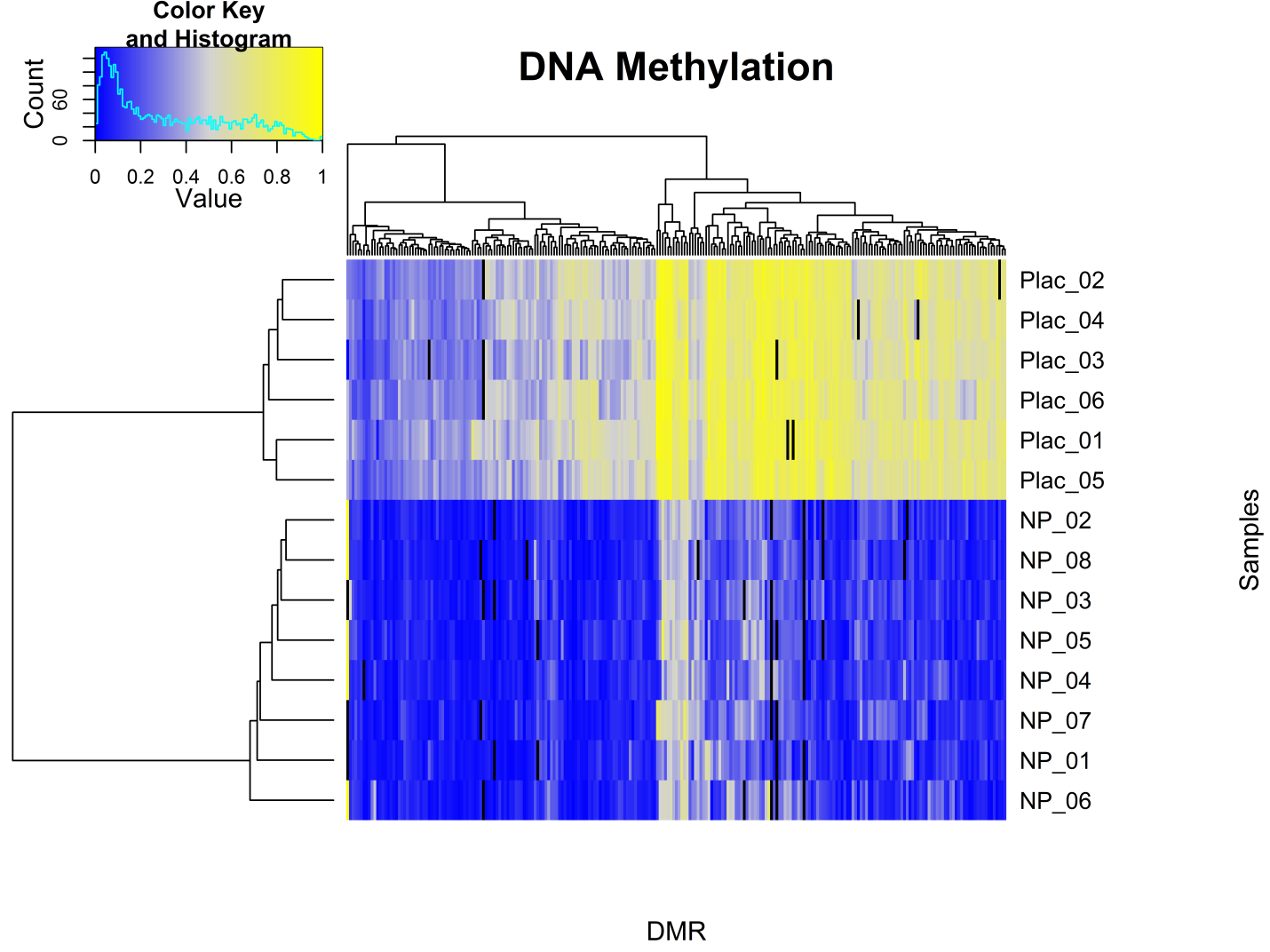
Supplementary Figure 10. MassARRAY confirmation of Differential Methylation.** Hierarchical clustering heatmap of the DNA methylation levels measured by MassARRAY within identified DMRs in placenta (Plac) and non-pregnant ccf DNA (NP). Only DMRs hypermethylated in placenta were selected. Blue=low CpG methylation; yellow=high CpG methylation; black=data missing or of insufficient quality.

**
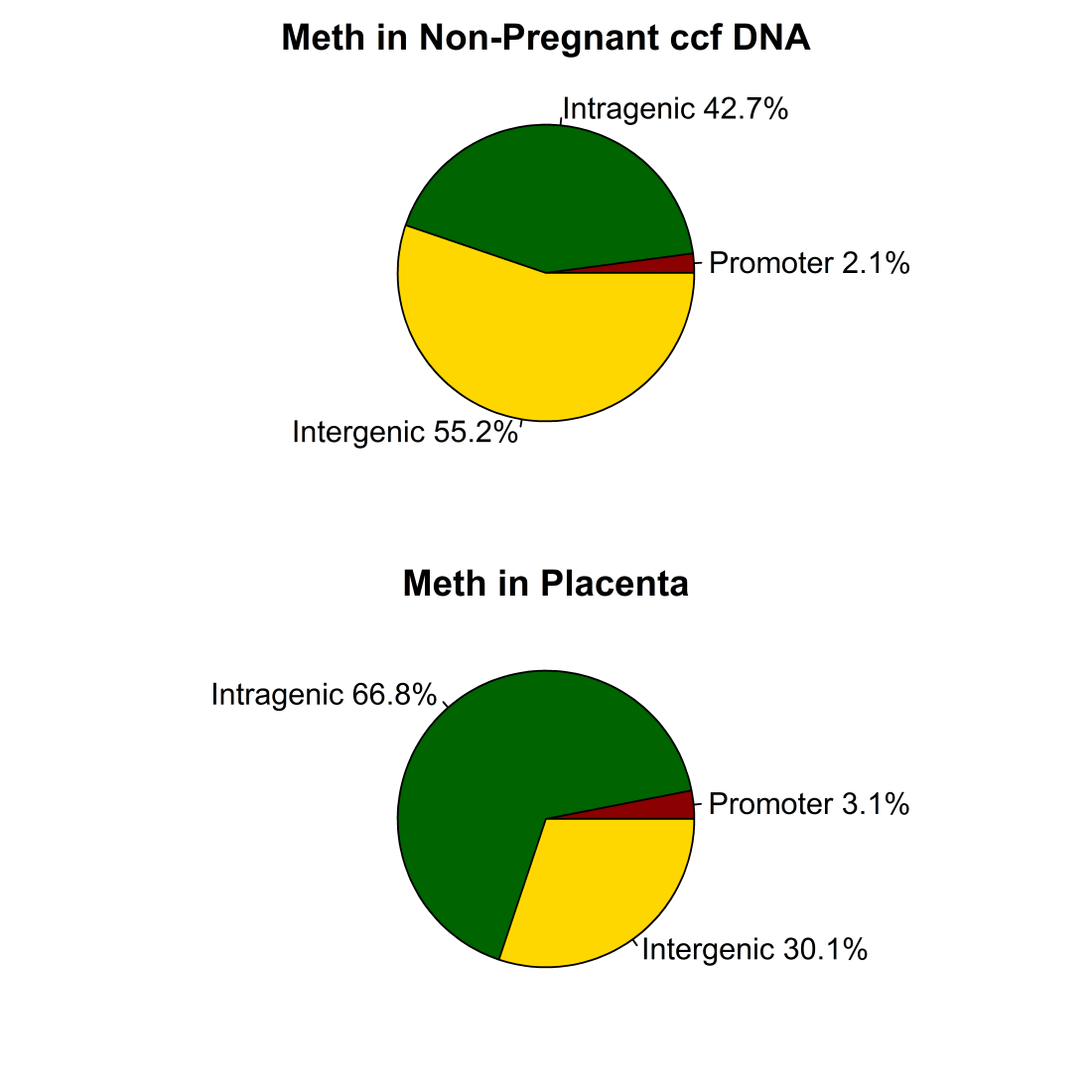
**

**Supplementary Figure 11. Distribution of DMRs between placenta and non-pregnant ccf DNA.** Pie charts display relative frequency of the location of DMRs hypermethylated in either non-pregnant ccf DNA or placenta. Intragenic=DMR overlaps with a known refseq gene; promoter=DMR overlaps with a gene promoter (1 kb upstream from each refseq gene); or intergenic=does not overlap with refseq gene or gene promoter.


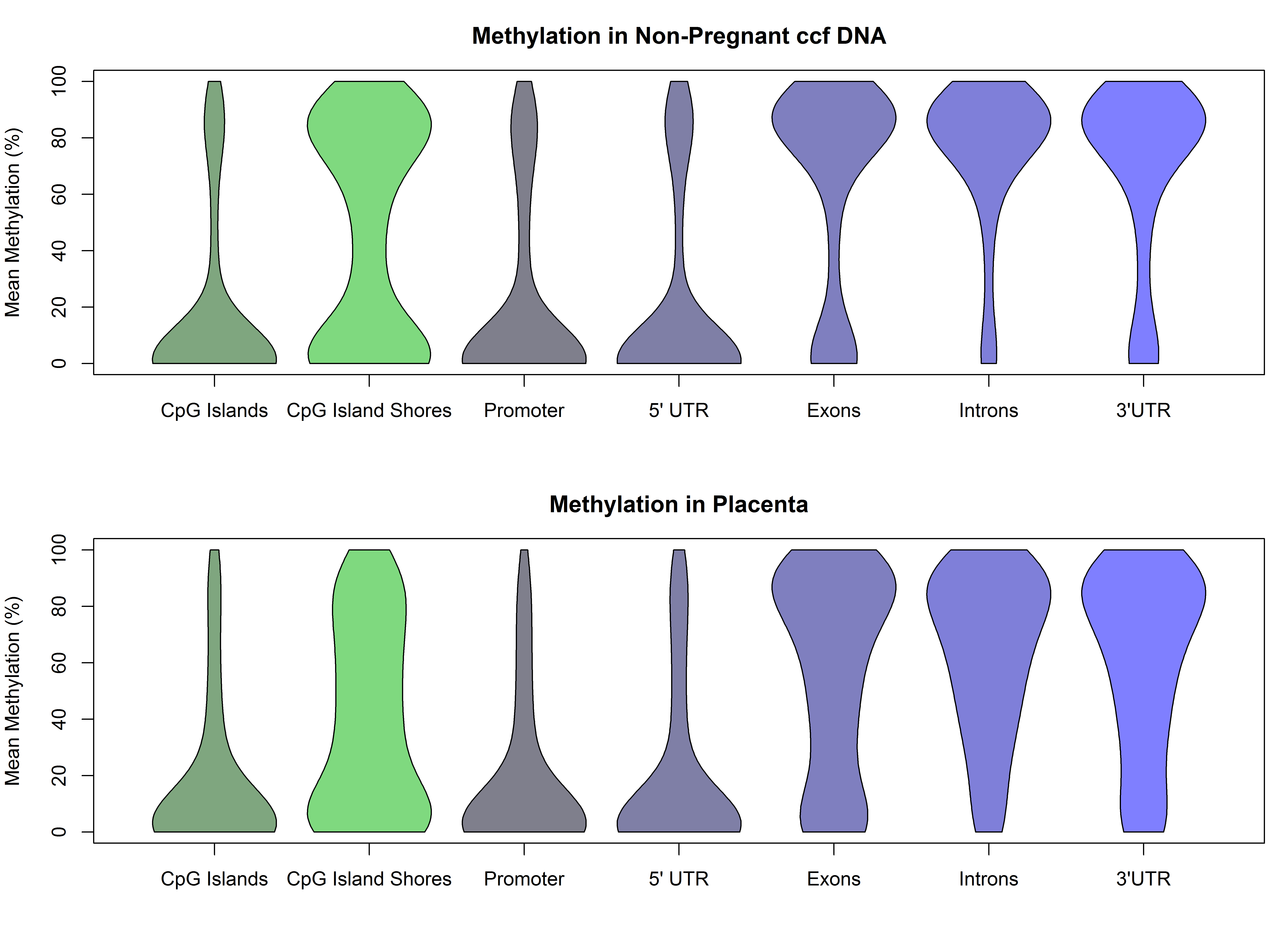


**Supplementary Figure 12. Distribution of DNA methylation levels in genomic regions.** Mean methylation was calculated in non-pregnant ccf DNA (Top) and placenta (Bottom) samples for all covered CpG sites within each of the listed genomic regions. The width of each violin plot describes the relative abundance of CpG sites with each methylation level within the listed regions.





**Supplementary Figure 13. Description of genomic location of DMRs when comparing buffy coat to placenta.** Each line represents the length of the corresponding chromosome. Regions more methylated in placenta (red) and buffy coat/PBMC (blue) shown. Distance from horizontal line for each chromosome is indicative of magnitude of differential methylation.

**
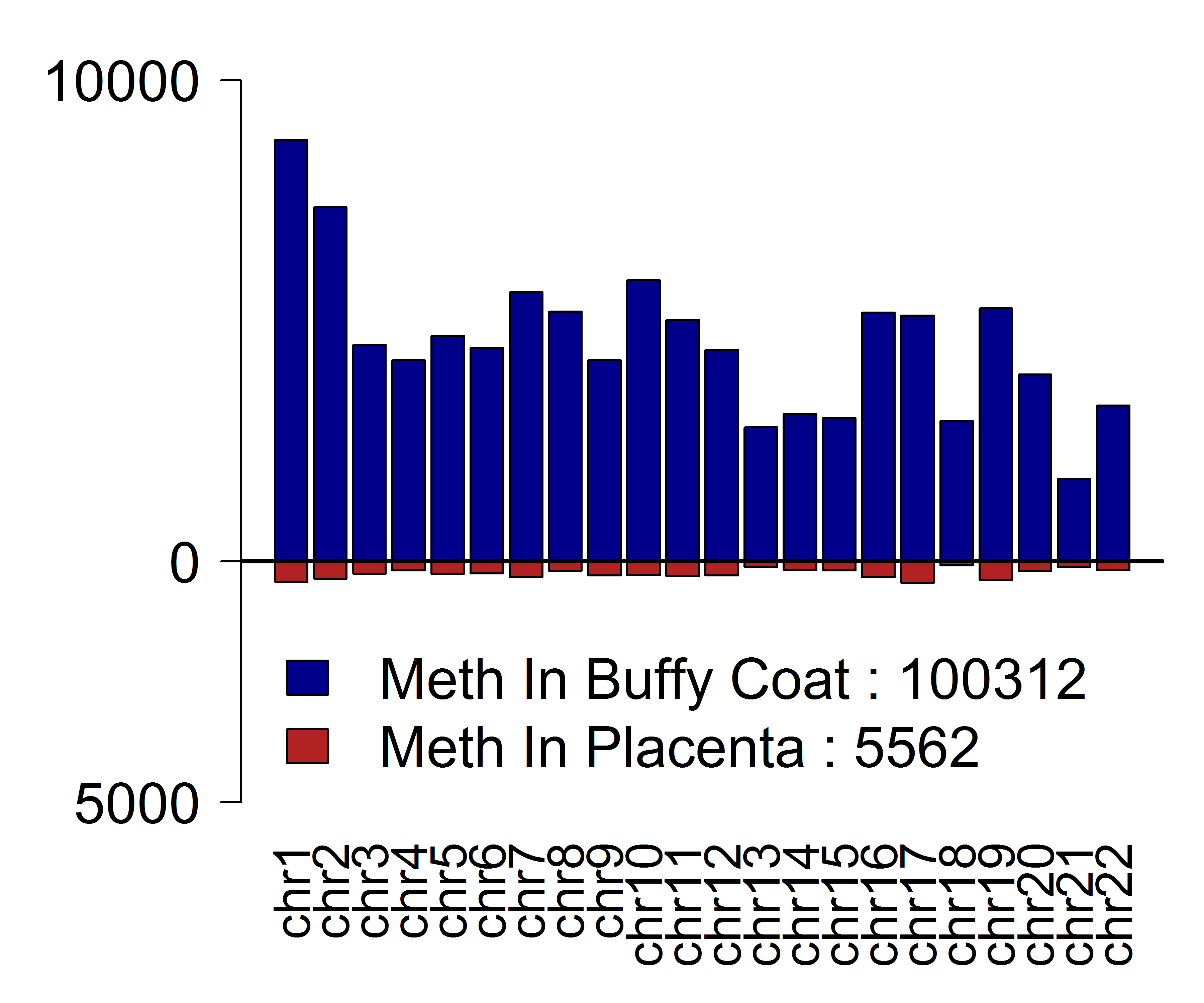
**

**Supplementary Figure 14. Description of the number of DMRs when comparing buffy coat to placenta.** Quantification of the number of DMRs per autosome. Regions more methylated in placenta (red) and buffy coat/PBMC (blue) shown.


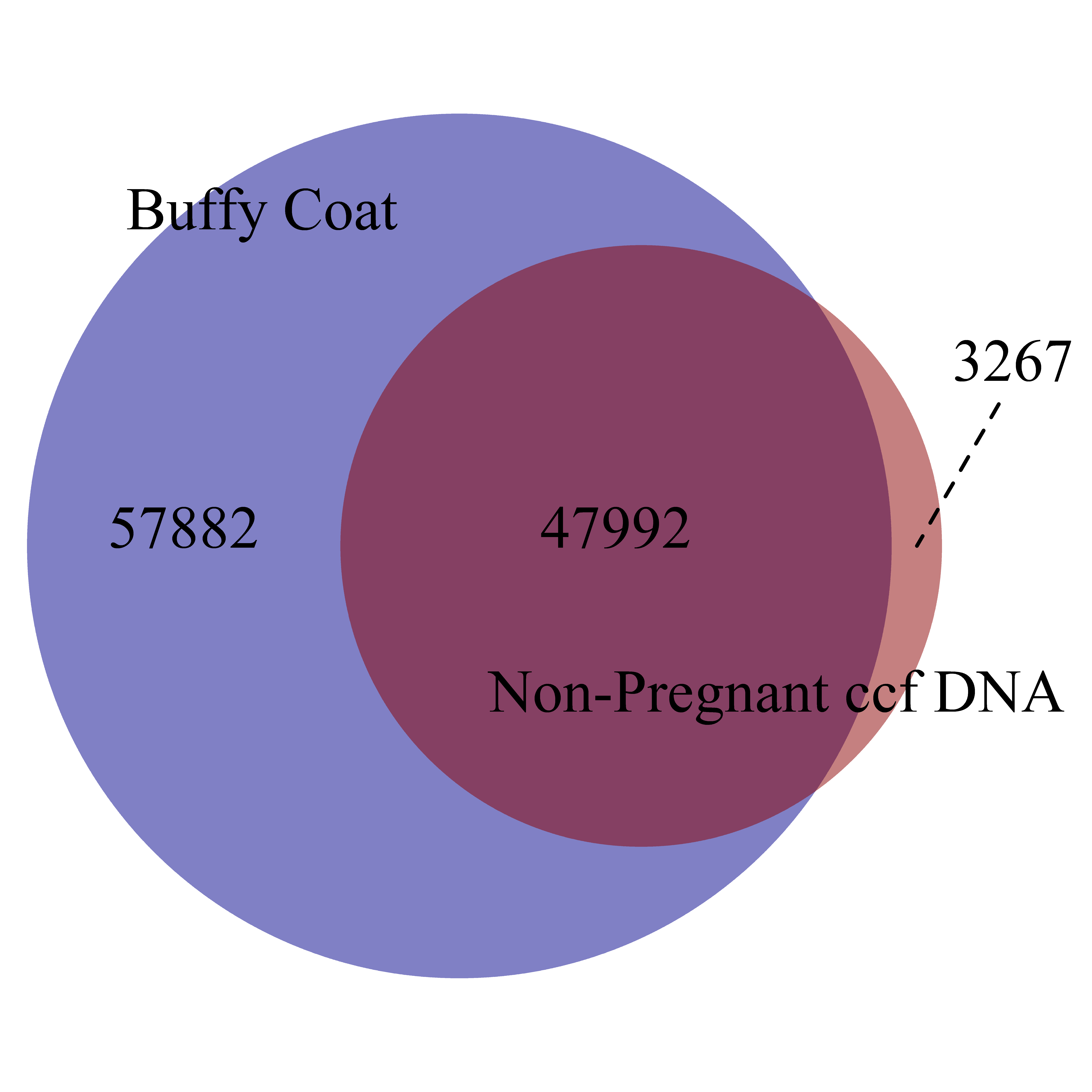


**Supplementary Figure 15.** **Venn diagram of DMR overlap.** Comparison of DMRs identified when comparing placenta to non-pregnant ccf DNA (red circle) or placenta to buffy coat (blue circle). The number of DMRs in each category is shown. The size of circles is proportionate to the number of identified DMRs within each group.


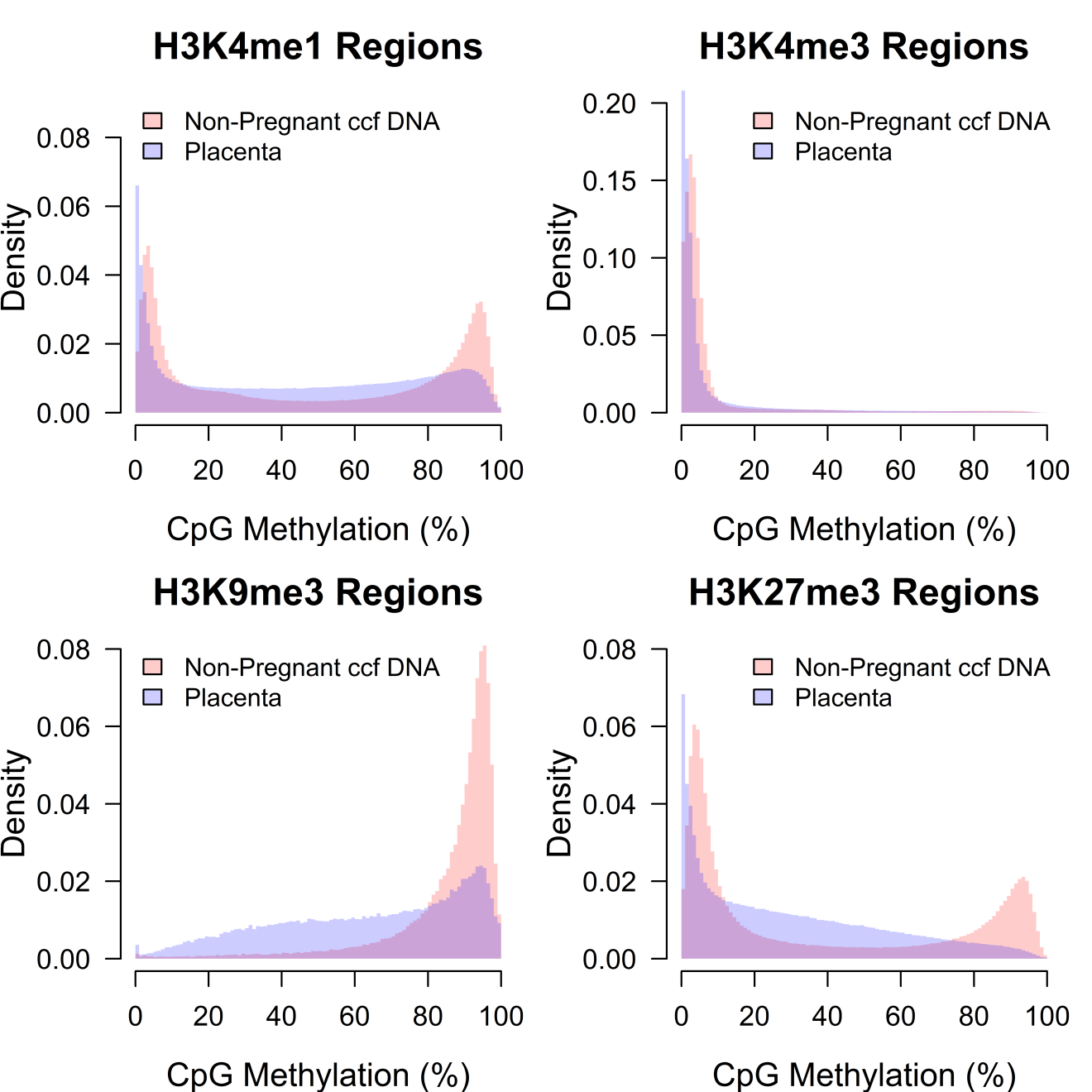


**Supplementary Figure 16.** **Linking differential DNA methylation to histone tail modifications in buffy coat/peripheral blood mononuclear cell (PBMC).** DNA methylation in both placenta and buffy coat/PBMC was assessed in areas defined during the ENCODE project as enriched in PBMC for histone H3 lysine 4 monomethylation (H3K4me1), histone H3 lysine 4 trimethylation (H3K4me3), histone H3 lysine-9 trimethylation (H3K9me3), and histone H3 lysine-27 trimethylation (H3K27me3). Red histograms are indicative of distribution of buffy coat CpG methylation within enriched regions; blue histograms are representative of CpG methylation within the same genomic regions in placenta. Note the high concordance of methylation patterns in H3K4me3 regions while there is general hypomethylation of H3K9me3 regions and lack of high levels of CpG methylation (80-100%) in H3K27me3 regions in placenta samples.


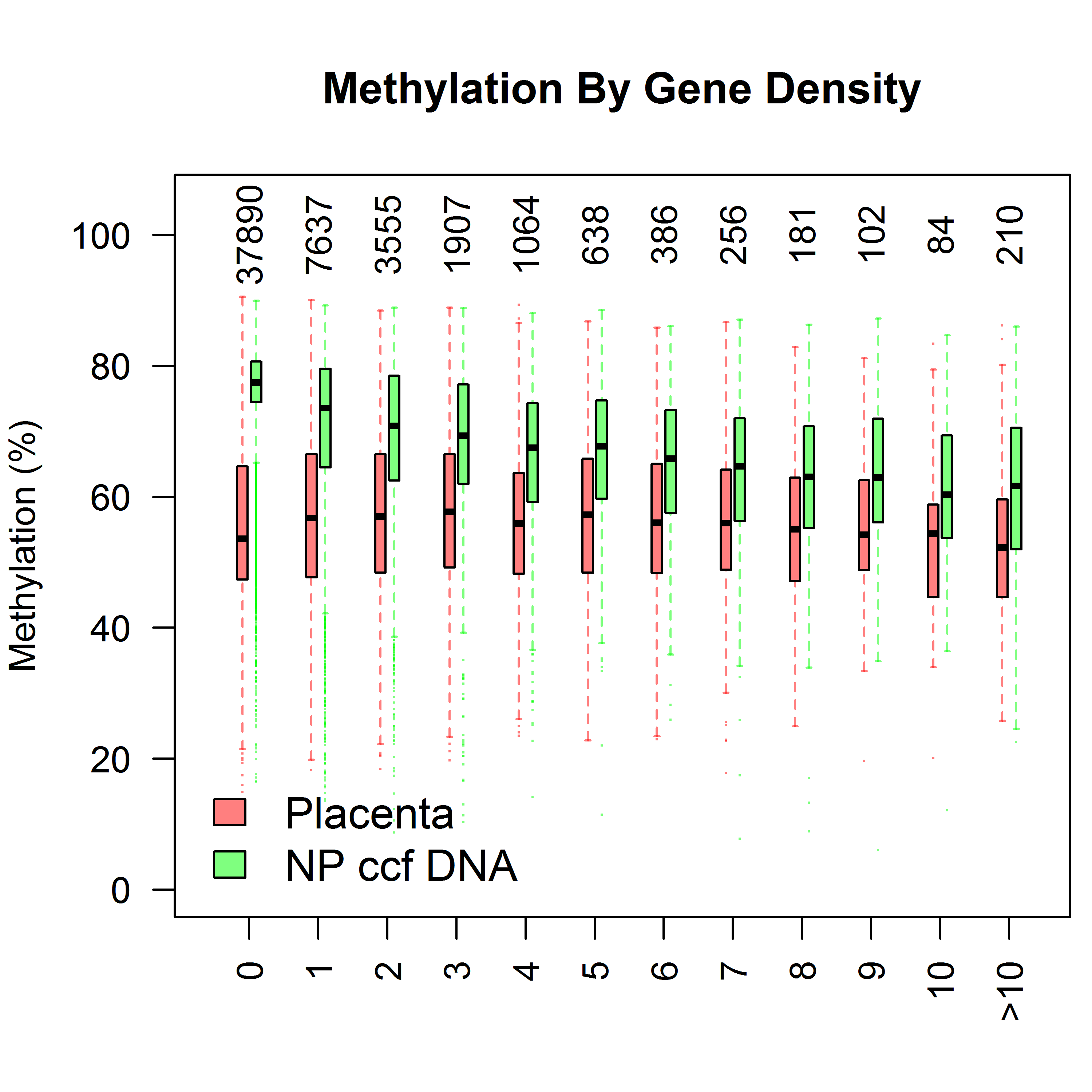


**Supplementary Figure 17.**Genomic methylation level by gene density at 50kbp bin level. Values on the x-axis represent the number of genes (calculated as the number of refseq transcription start sites) per 50kbp bin. Numbers along the top indicate the number of genomic bins analyzed.


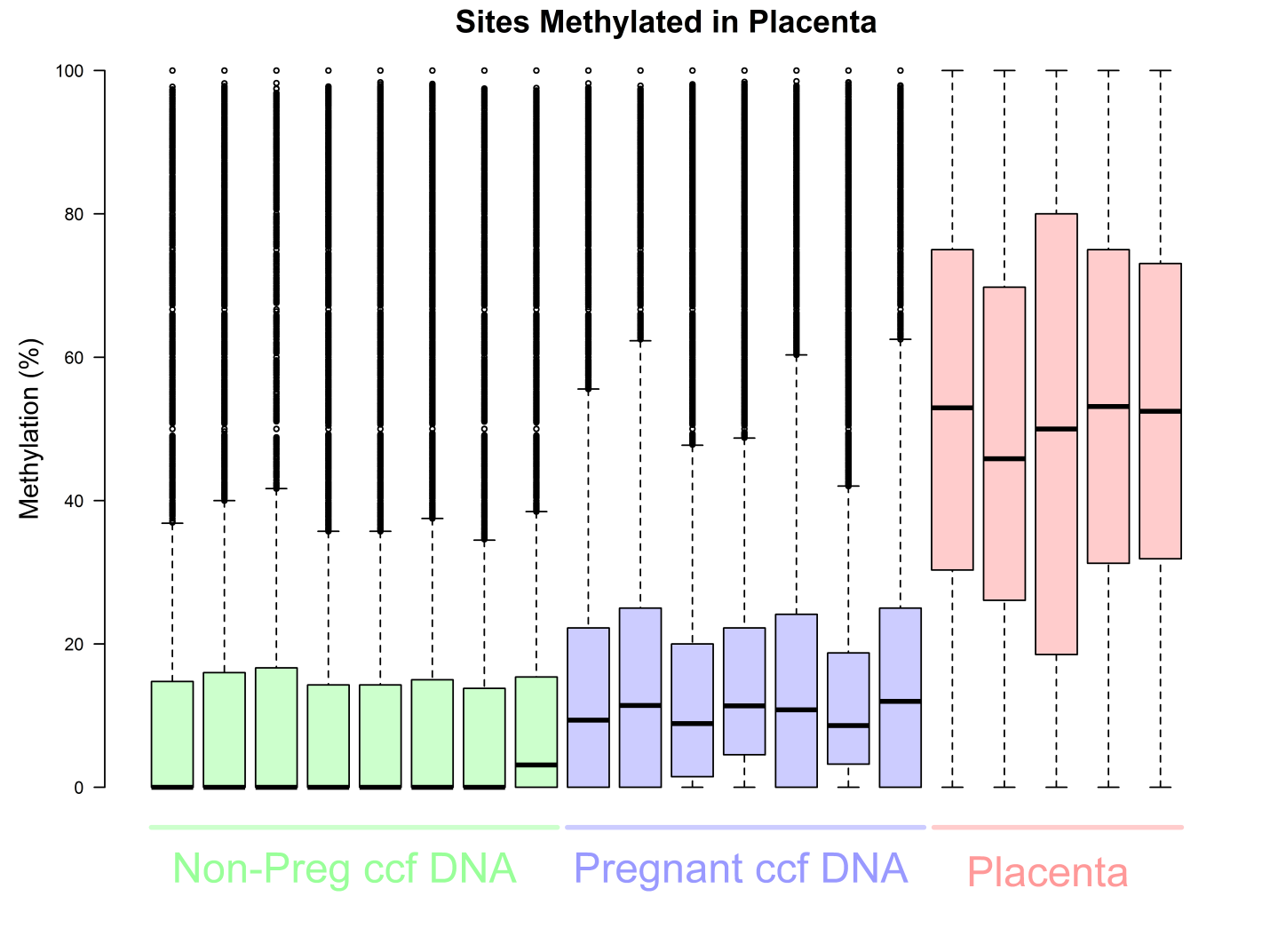


**Supplementary Figure 18. CpG methylation in placenta hypermethylated regions.** Box plots represent methylation of all cytosines located within DMRs hypermethylated in placenta tissue relative to non-pregnant ccf DNA. Each sample is represented by a single box plot (n=8 non-pregnant ccf DNA; n=7 pregnant ccf DNA samples; n=5 placenta samples). Note the increase of the median methylation level (shown as the solid bar in each box plot) in pregnant ccf DNA relative to non-pregnant ccf DNA.


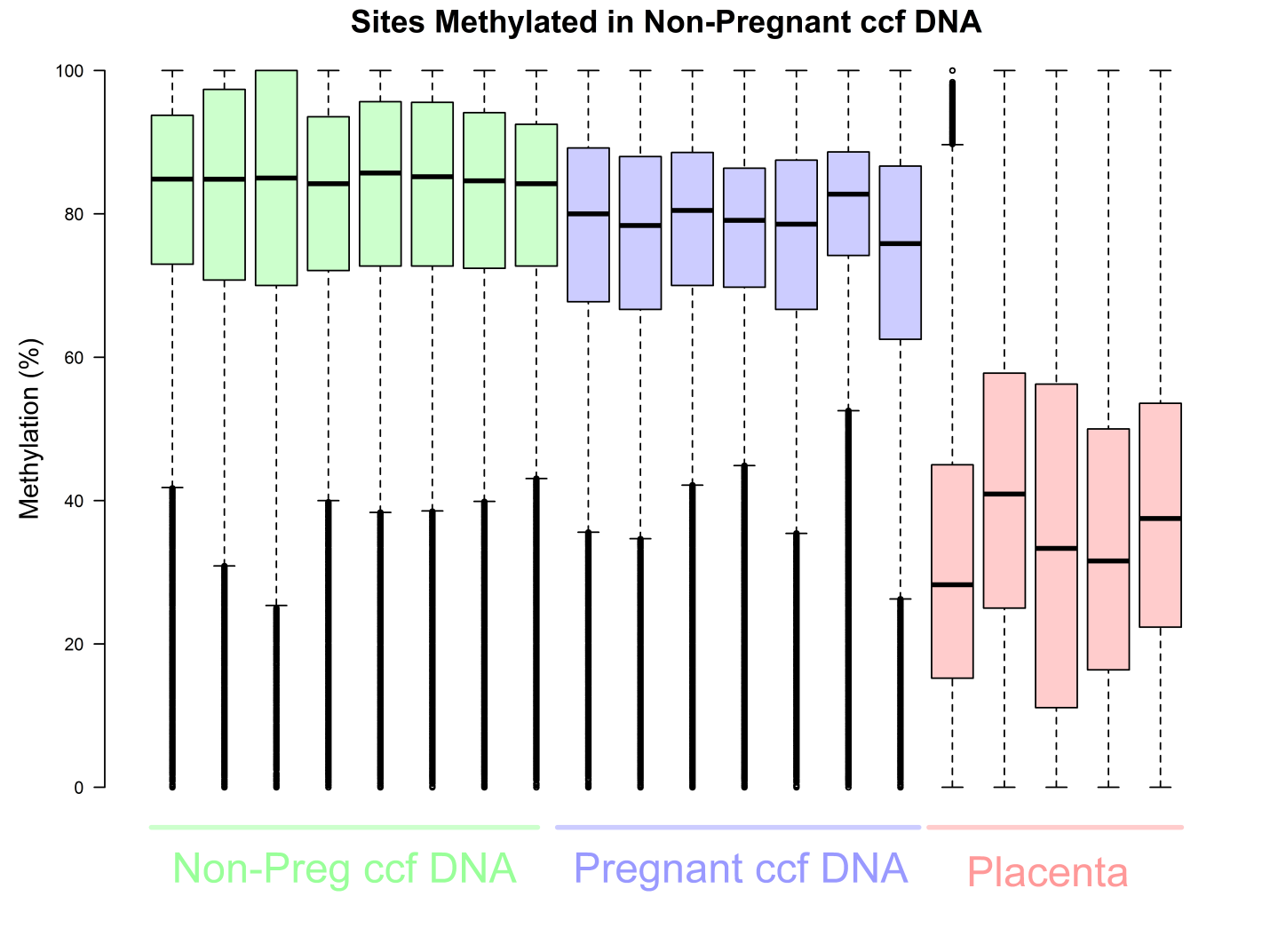


**Supplementary Figure 19. CpG methylation in non-pregnant ccf DNA hypermethylated regions.** Box plots represent methylation of all cytosines located within DMRs hypermethylated in non-pregnant ccf DNA relative to placenta. Each sample is represented by a single box plot (n=8 non-pregnant ccf DNA; n=7 pregnant ccf DNA samples; n=5 placenta samples). Note the decrease of the median methylation level (shown as the solid bar in each box plot) in pregnant ccf DNA relative to non-pregnant ccf DNA.


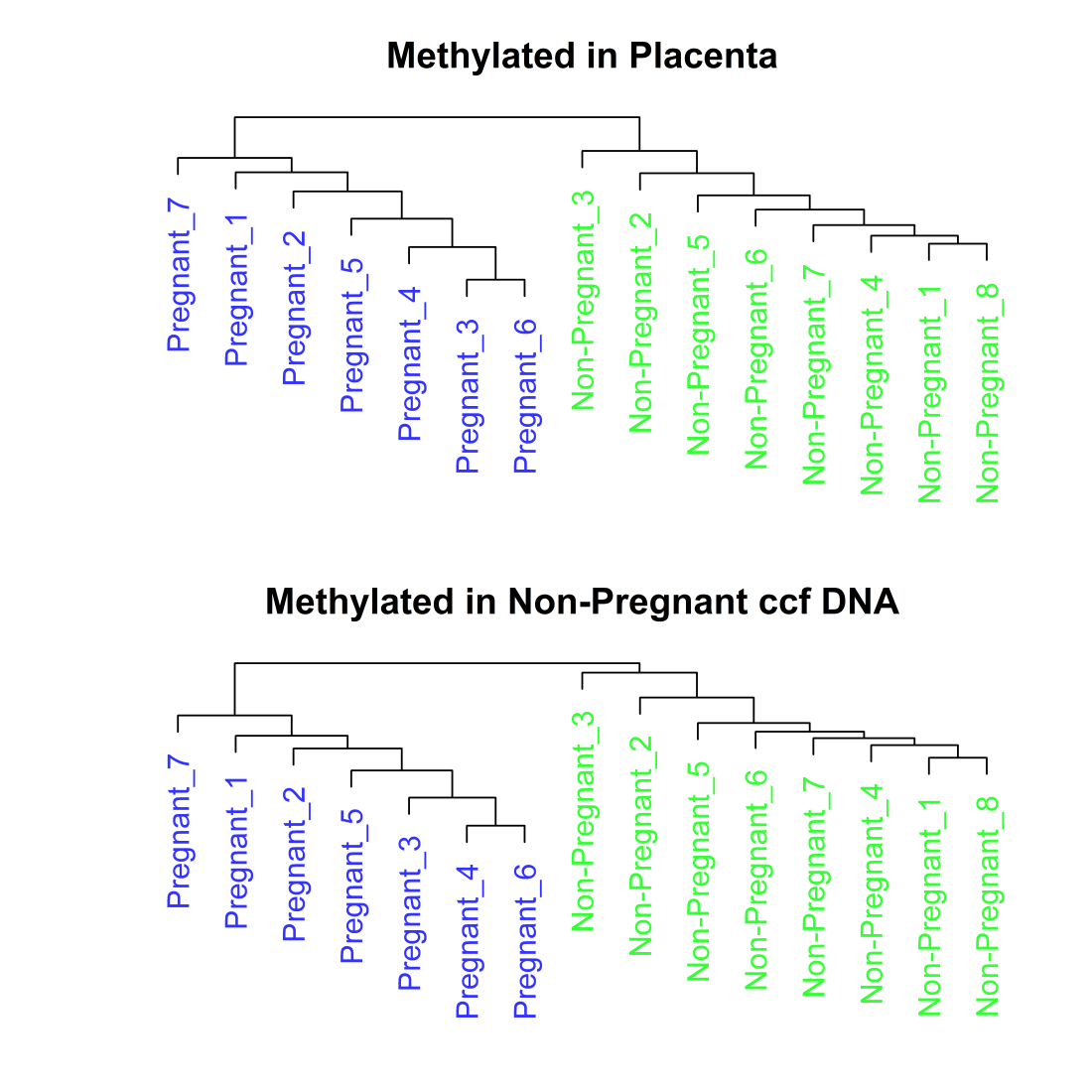


**Supplementary Figure 20.** Top) Hierarchical clustering of ccf DNA using methylation of placenta-specific methylated regions. Bottom) Hierarchical clustering of ccf DNA using methylation of non-pregnant ccf DNA specific methylated regions.





**Supplementary Figure 21.** Ratio of methylated CpG, CHG, and CHH cytosines within large fragments (>200bp) relative to methylated cytosines in small fragments (<200bp) after scaling for number of cytosines measured. Boxplots represent the value for each sample within a particular sample group.


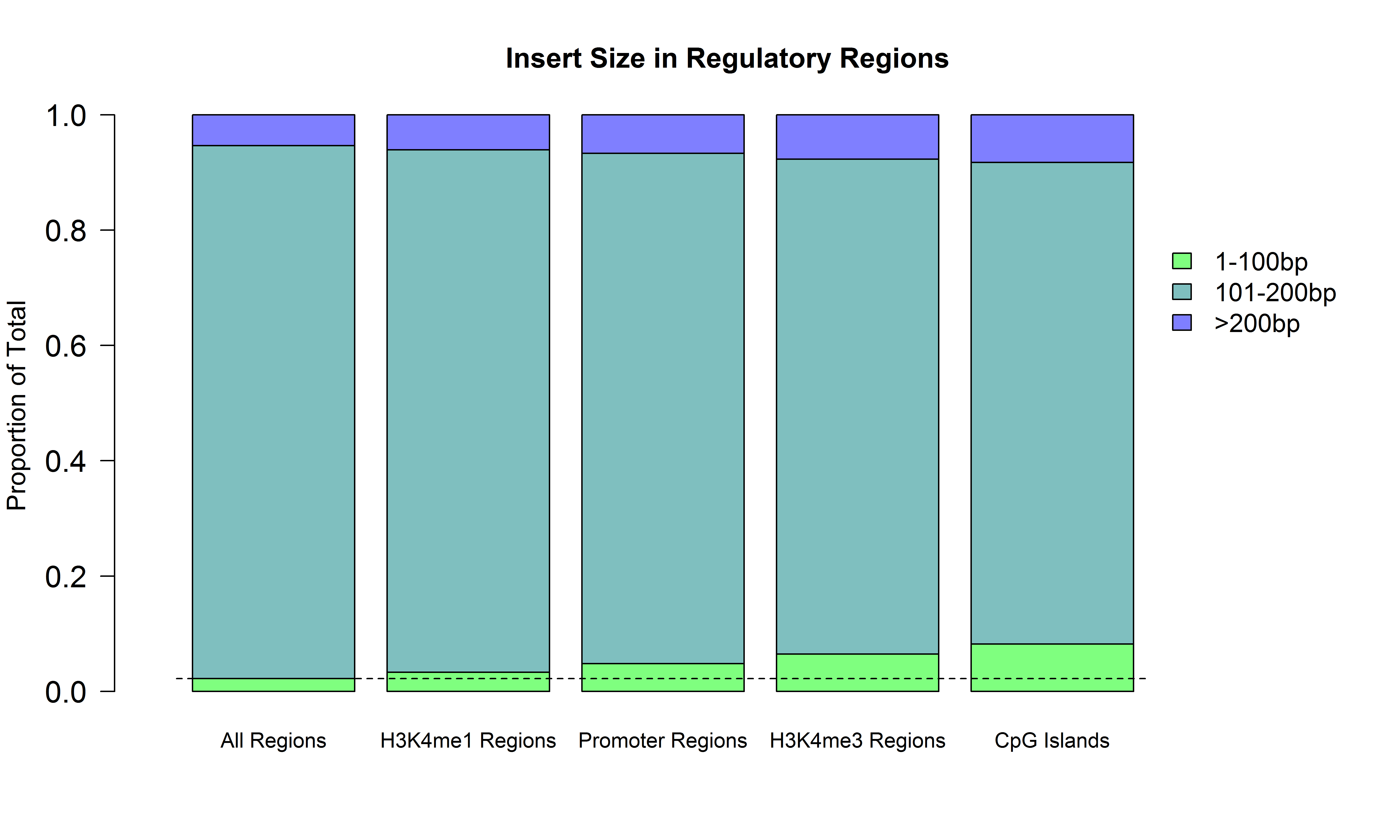


**Supplementary Figure 22.** Ratio of methylated CpG, CHG, and CHH cytosines within large fragments (>200bp) relative to methylated cytosines in small fragments (<200bp) after scaling for number of cytosines measured. Boxplots represent the value for each sample within a particular sample group.


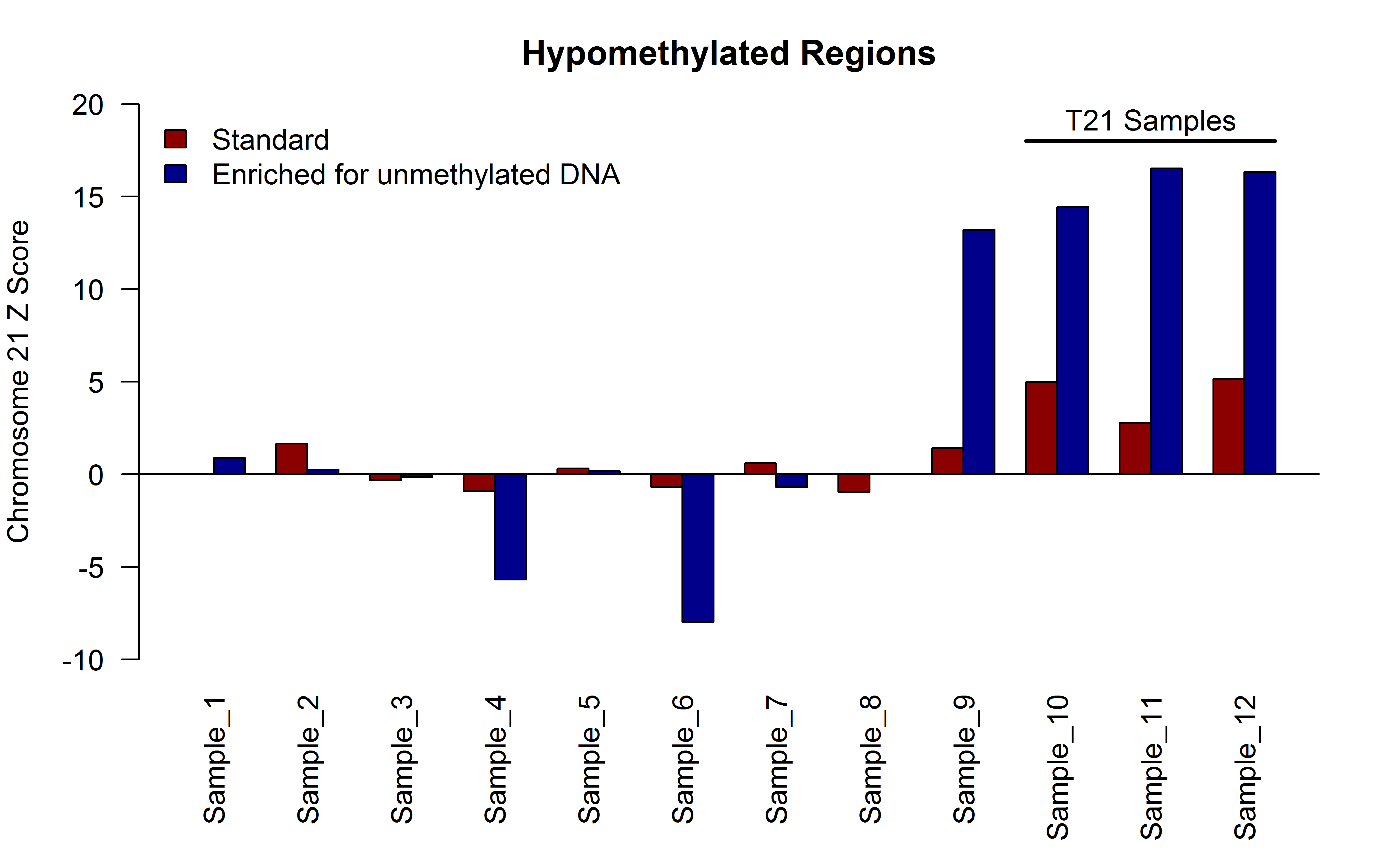


**Supplementary Figure 23. Enrichment for hypomethylated DNA enhances aneuploidy detection in trisomy 21 (T21) samples.** Barplot shows the chromosome 21 z-score for each of 12 ccf DNA samples obtained from pregnant female donors (9 euploid, 3 T21) and subjected to massively parallel sequencing using either all input ccf DNA (Standard; red) or only the unmethylated DNA fraction obtained after depletion of the methylated fragments by MCIp (enriched for unmethylated DNA; blue).
