# Supplementary figures and images for: Whole genome bisulfite sequencing of cell-free DNA and its cellular contributors uncovers placenta hypomethylated domains
Source: Genome Biol. 2015 Apr 15;16(1):78. doi: 10.1186/s13059-015-0645-x (PMC4427941; doi:10.1186/s13059-015-0645-x)

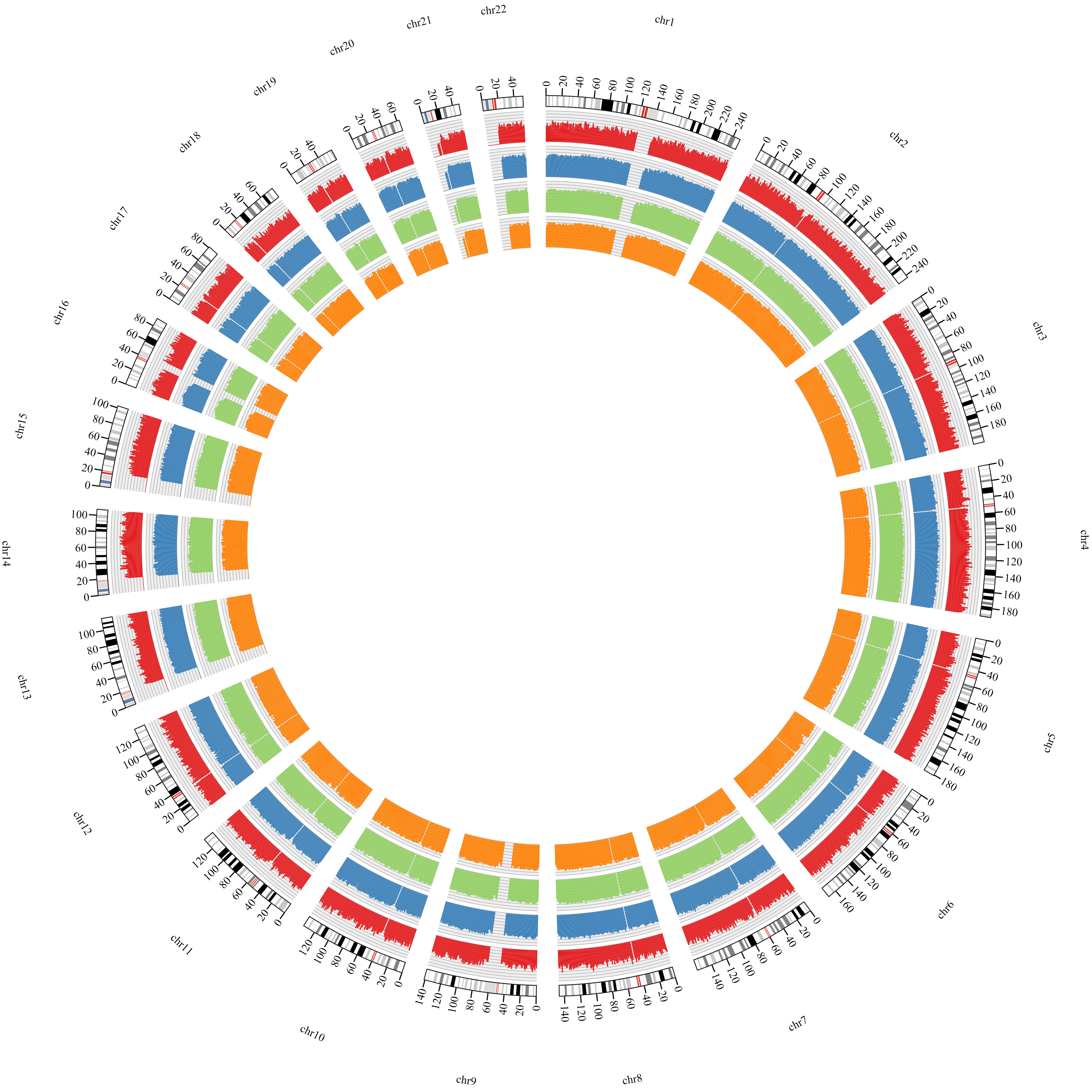

Supplement: Additional file 2: Figure S2. — Circos plot of all sample types. All autosomes are plotted and labeled along the perimeter with each concentric circle representing the mean methylation for each sample type. From the outside of the concentric circles working inward, methylation levels are shown for placenta (red), pregnant ccf DNA (blue), non-pregnant ccf DNA (green), and buffy coat (orange). The height of each histogram within each concentric circcle represents the mean methylation level for CpG sites within non-overlapping, 1 MB genomic bins. [file 13059_2015_645_MOESM2_ESM.png]
